# Branched-Chain Fatty Acids Alter the Expression of Genes Responsible for Lipid Synthesis and Inflammation in Human Adipose Cells

**DOI:** 10.3390/nu14112310

**Published:** 2022-05-31

**Authors:** Aleksandra Czumaj, Tomasz Śledziński, Adriana Mika

**Affiliations:** Department of Pharmaceutical Biochemistry, Faculty of Pharmacy, Medical University of Gdansk, Debinki, 80-211 Gdansk, Poland; tomasz.sledzinski@gumed.edu.pl (T.Ś.); adriana.mika@gumed.edu.pl (A.M.)

**Keywords:** iso-BCFA, anteiso-BCFA, branched-chain fatty acids, adipocytes, obesity, inflammation

## Abstract

Recently, we have demonstrated a decreased level of iso-branched-chain fatty acids (iso-BCFAs) in patients with excessive weight. However, it is still unclear whether BCFAs may influence lipid metabolism and inflammation in lipogenic tissues. To verify this, human visceral adipocytes were cultured with three different concentrations of selected iso-BCFA (14-methylpentadecanoic acid) and anteiso-BCFA (12-methyltetradecanoic acid), and then the expression of genes associated with lipid metabolism (*FASN*—fatty acid synthase; *SREBP1*—sterol regulatory element-binding protein 1; *SCD1*—stearoyl-CoA desaturase; *ELOVL4*—fatty acid elongase 4; *ELOVL6*—fatty acid elongase 6; *FADS2*—fatty acid desaturase 2; *FADS1*–fatty acid desaturase 1) and inflammation (*COX-2*—cyclooxygenase 2; *ALOX-15*—lipoxygenase 15; *IL-6*—interleukin 6) were determined. This study demonstrates for the first time that incubation with iso-BCFA decreases the expression of adipocyte genes that are associated with lipid metabolism (except *FASN*) and inflammation. These findings suggest that changes in the iso-BCFA profile in obese patients may contribute to adipose inflammation and dyslipidemia. Further studies should evaluate whether iso-BCFA supplementation in obese patients would be beneficial.

## 1. Introduction

Obesity is a complex chronic disease that adversely affects nearly all physiological functions of the body. It contributes to reduced life expectancy, impaired quality of life, and increases the risk of developing multiple disease conditions, such as type 2 diabetes (T2D); non-alcoholic fatty liver disease (NAFLD); hypertension; coronary heart disease; stroke; and several types of cancer [1,2,3,4]. The well-known hallmark of obesity is dyslipidemia [5,6]. One group of fatty acids that is gaining research interest in this matter is branched-chain fatty acids (BCFAs). BCFAs are a class of mostly saturated fatty acids with one or more methyl branches in their carbon chains. Based on branch point position, the following types are distinguished: iso-BCFA (with a methyl branch on the penultimate carbon, i.e., one from the end) and anteiso-BCFA (with the methyl branch located on the antepenultimate carbon, i.e., two from the end), (Table 1) [7,8]. Until now, in humans, BCFAs have been found in vermix caseosa, breast milk, adipose tissue, and serum [9,10,11,12]. Moreover, there is growing evidence that BCFAs are associated with obesity and inflammation. In our previous studies, we showed that in the serum of obese patients the levels of BCFAs, especially iso-BCFA, were lower in comparison to non-obese patients [12,13]. Other authors also reported similar results but in adipose tissue [11]. However, the consequences of these changes are still not explored. Moreover, the anti-inflammatory properties of BCFA were mostly studied in the context of dietary BCFAs and gastrointestinal health [14,15,16]. In the present study we focus on adipocyte inflammation since several researchers have confirmed that low-grade inflammation of adipose tissue is associated with obesity-related metabolic diseases [17,18,19,20].

We also found statistically significant inverse correlations between the serum concentration of iso-BCFA and triglycerides (TG), as well as C-reactive protein (marker of inflammation) in obese patients [12]. However, in our previous research, we only speculated about a possible molecular mechanism of this relationships. In this paper, we investigated whether changes in BCFA level are just another disorder associated with dyslipidemia and inflammation observed in obese patients or, if these BCFA alterations may play a role in the development of dyslipidemia and inflammation by affecting the adipocytes, one of the main types of cells involved in lipid metabolism and inflammation in humans.

The aim of this study was to analyze the effect of selected BCFAs on the expression of genes related to lipid synthesis and inflammation in adipocytes.

## 2. Materials and Methods

### 2.1. Cell Culture and Treatment

We used primary human white preadipocytes that were isolated from adult visceral adipose tissue. The cells, all media and supplements were purchased from PromoCell (PromoCell GmbH, Heidelberg, Germany). The cells were cultured and differentiated according to the manufacturer’s instructions. In brief, the preadipocytes were plated with a plating density of 5000 cells per cm^2^ on 6-well plates and cultured in preadipocyte basal medium supplemented with fetal calf serum (final concentration: 0.05 mL/mL); endothelial cell growth supplement (0.004 mL/mL); recombinant human epidermal growth factor (10 ng/mL); hydrocortisone (1 μg/mL); and heparin (90 μg/mL). After the cells reached total confluency stage, preadipocyte growth medium was replaced by preadipocyte differentiation medium for 72 hours. Preadipocyte differentiation medium was prepared from basal medium; d-Biotin (8 μg/mL); recombinant human insulin (0.5 μg/mL); dexamethasone (400 ng/mL); IBMX (44 μg/mL); L-thyroxine (9 ng/mL); and ciglitazone (3 μg/mL). After 72 h, preadipocyte differentiation medium was replaced by adipocyte nutrition medium (adipocytes basal medium supplemented with fetal calf serum (0.03 mL/mL); d-Biotin 8 (μg/mL); recombinant human insulin (0.5 μg/mL); and dexamethasone (400 ng/mL). The medium was changed every 2 days. After 2 weeks the differentiation process was complete and only mature adipocytes were present on the plates. Adipocytes differentiation was confirmed by oil red O staining. All cells were cultured in a humidified atmosphere of 95% air and 5% CO_2_ at 37 °C.

The mature visceral adipocytes were incubated with 14-methylpentadecanoic acid (iso-palmitic acid, iso 16:0, 14-MPA) or 12-methyltetradecanoic (anteiso-pentadecanoic acid, anteiso 15:0, 12-MTA). These specific BCFAs were selected based on our previous research. The 14-MPA was selected because it was a branched-chain fatty acid whose serum content significantly statistically differed between patients with excess weight and healthy subjects with normal weight. The 12-MTA was selected based on similar serum levels in obese patients and a similar length of the carbon chain to the 14-MPA. Due to the similar serum contents of both BCFAs, we were able to use the same experimental concentrations. The abovementioned BCFAs were used in three different concentrations: 2 µM, 5 µM, and 10 µM for 48 h. The concentrations were selected to mimic normal physiological conditions (5 µM) and states of decreased and increased BCFA concentrations, 2 µM and 10 µM, respectively. BCFAs were purchased from Sigma-Aldrich (St. Louis, MO, USA). Adipocytes in basal adipocyte nutrition medium were used as a control. The selected concentrations of BCFAs did not influence the cells’ viability when assessed by MTT assay. Due to the limited possible number of passages of primary human white preadipocytes, the number of experiments that can be performed with these cells is limited. We used whole material that was obtained from purchased cells to perform the experiments presented in this paper.

### 2.2. Real-Time PCR Analysis of mRNA Levels

Cells were lysed directly on the culture plate with the QIAzol Lysis Reagent (Qiagen) after medium removing and PBS washing. The total RNA was isolated from the cells with an RNeasy Lipid Tissue Mini Kit (Qiagen, Hilden, Germany). RNA quantity and purity were determined by optical density and A260/280 and A260/230 ratio using a NanoDrop One Microvolume UV-Vis Spectrophotometer (Thermo Fisher Scientific, Waltham, MA, USA). The integrity of RNA was assessed by RNA StdSens Assay on an Experion Automated Electrophoresis System (Bio-Rad Laboratories, Hercules, CA, USA). An amount of 1 µg of total RNA was reverse transcribed using a RevertAid First Strand cDNA Synthesis Kit (Thermo Fisher Scientific, Waltham, MA, USA)). Real-time PCR was performed on a CFX Connect Real-Time PCR Detection System (Bio-Rad) using a SensiFAST SYBR No-ROX Kit (Meridian Bioscience, Cincinnati, OH, USA). All primers were synthesized by Genomed S.A. (Warsaw, Poland). The specificity of the mRNA amplification was confirmed by melting curve analysis. Real-time PCR data were analyzed using the 2^−^^△△dCt^ relative quantification method.

### 2.3. Statistical Analysis

All data are presented as mean ± SD. All experimental conditions were analyzed in triplicates in three independent experiments. The statistical significance of the differences between the experimental and control conditions was verified with the Mann–Whitney U test. The results were considered significant for *p*-values < 0.05. All analyses were conducted using Statistica 13 (StatSoft, Cracow, Poland).

## 3. Results and Discussion

**Figure 1:**
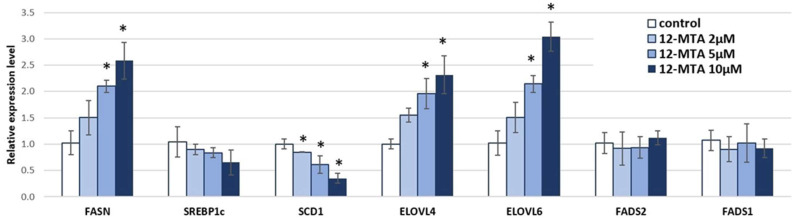
Relative expression level of selected genes involved in fatty acid synthesis and metabolism in visceral adipocytes incubated for 48 h in different concentrations of 12-methyltetradecanoic (12-MTA). Data are presented as mean ± SD. * *p* < 0.05 compared to control. *FASN*—fatty acid synthase; *SREBP1*—sterol regulatory element-binding protein 1; *SCD1*—stearoyl-CoA desaturase; *ELOVL4*—fatty acid elongase 4; *ELOVL6*—fatty acid elongase 6; *FADS2*—fatty acid desaturase 2; *FADS1*—fatty acid desaturase 1.

Incubation with 12-MTA increased the expression of adipocytes genes encoding enzymes involved in fatty acid synthesis (*FASN*); elongation of polyunsaturated fatty acids (PUFA)–*ELOVL4*; and elongation of saturated (SFA) and monounsaturated fatty acids (MUFA)–*ELOVL6*. In contrast, the relative expression level of gene encoding stearoyl-CoA desaturase (*SCD1*) was decreased. The 12-MTA did not change the expression level of genes encoding fatty acid desaturases involved in PUFA metabolism (*FADS1*, *FADS2*), (Figure 1).

**Figure 2:**
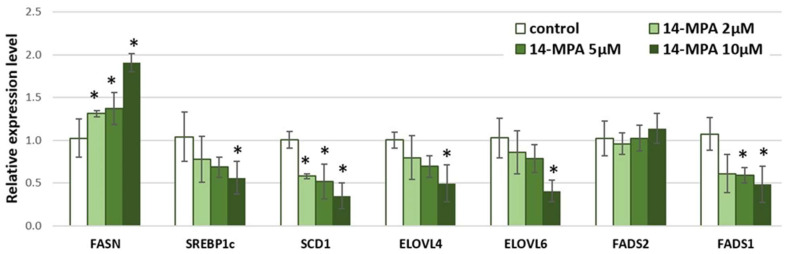
Relative expression level of selected genes involved in fatty acid synthesis and metabolism in visceral adipocytes incubated for 48 h in different concentrations of 14-methylpentadecanoic acid (14-MPA). Data are presented as mean ± SD. * *p* < 0.05 compared to control. *FASN*—fatty acid synthase; *SREBP1*—sterol regulatory element-binding protein 1; *SCD1*—stearoyl-CoA desaturase; *ELOVL4*—fatty acid elongase 4; *ELOVL6*—fatty acid elongase 6; *FADS2*—fatty acid desaturase 2; *FADS1*—fatty acid desaturase 1.

Incubation with 14-MPA increased only the expression of gene-encoding FASN. In contrast to 12-MTA, 14-MPA had the opposite effect on the expression level of genes encoding *ELOV4* and *ELOV6*. The decreased expression level was observed for genes encoding sterol regulatory element-binding protein 1 (*SREBP1c*) and *FADS1* (Figure 2). Similar to 12-MTA, 14-MPA did not change the expression of *FADS2*.

In vivo and in vitro studies have shown that in adipose tissue SCD1 is involved in the promotion of lipid mobilization by enhancing lipolysis and lipogenesis [21,22]. SCD1 is a key enzyme involved in de novo lipogenesis and is responsible for the conversion of SFAs to MUFAs. MUFAs such as, for example, palmitoleate (16:1) and oleate (18:1) can then be used for TG synthesis. Thus, changes in SCD1 activity/expression are related to TG level [23]. Furthermore, studies have shown that oleic acid, a product of SCD1, may upregulate the expression of adipose TG lipase (*ATGL*) and hormone-sensitive lipase (*HSL*) in adipose tissue [24,25,26]. Therefore, the increased expression of SCD1 may lead to enhancing the release of free fatty acids (FFAs) from adipose tissue. These FFAs can be re-esterified in adipose tissue to form TG. Moreover, in obese subjects, a higher mRNA level of SCD1 was observed in comparison to non-obese subjects [27]. Therefore, an increased production and release of FFAs from adipose tissue can contribute to dyslipidemia that is observed in obese subjects. We show in this study that both iso- and anteiso-BCFAs are able to decrease the expression level of *SCD1*; however, iso-BCFAs are decreased in obese subjects [12]. SREBP1c is a well-known transcription factor that activates genes involved in FA and TG synthesis, including SCD1 [28,29]. Decrease in *SREBP1c* mRNA level suggest a possible molecular mechanism by which iso-BFCAs can influence *SCD1* gene expression and lipid metabolism.

We observed that under the influence of BCFA, the mRNA levels of *SCD1* and *FASN* changed in the opposite way. Although *FASN* can be also regulated by SREBP1 [30], our results suggest that in adipocytes, *FASN* expression after BCFA treatment may be regulated in an SPEBP1-independent manner [31]. Opposite changes in the expression level of these lipogenesis genes (*SCD1* and *FASN*) were also observed by Eissing et al. [27] who observed that in the visceral white adipose tissue of obese subjects, mRNA levels of *FASN* were lower and *SCD1* mRNA were higher in comparison to non-obese subjects [27].

Studies have shown that *ELOVL6* has a crucial role in the development of obesity-induced pathologies. Mice with *ELOVL6* deficiency were protected from hyperinsulinemia, hyperglycemia, and hyperleptinemia [32,33,34]. In this study, we demonstrated that iso-BCFA has the ability to decrease the expression level of *ELOVL6*. Since the concentration of this type of BCFAs is lower in the serum of obese subjects, it can be speculated that supplementation of iso-BCFAs may lead to the improvement of insulin sensitivity in obese subjects.

**Figure 3:**
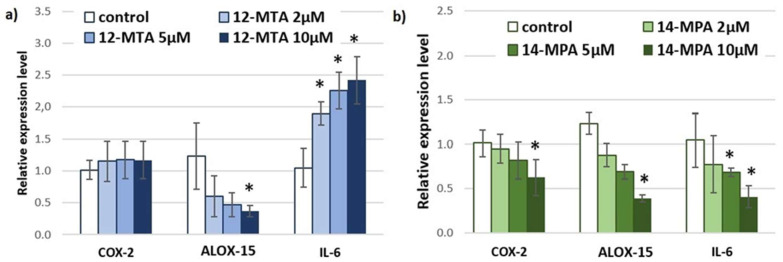
Relative expression level of selected genes involved in inflammation in visceral adipocytes incubated for 48h in different concentrations of (**a**) 12-methyltetradecanoic (12-MTA) and (**b**) 14-methylpentadecanoic acid (14-MPA). Data are presented as mean ± SD. * *p* < 0.05 compared to control. *COX-2*—cyclooxygenase 2; *ALOX-15*—lipoxygenase 15; *IL-6*—interleukin 6.

BCFAs can also alter the expression of the genes that are involved in inflammation (Figure 3a,b). The greatest effect was observed for interleukin 6 (*IL-6*). Interestingly, iso-BCFA and anteiso-BCFA had the opposite effect on the expression of this gene. The 14-MPA, a representative of iso-BCFA, decreased the expression of *IL-6* in the dose-dependent matter (Figure 3b). However, this type of BCFA is decreased in subjects with obesity. IL-6 is a major inflammatory mediator involved in obesity-related chronic inflammation, which may result in an increased risk of cardiovascular complication, insulin resistance, and type 2 diabetes [35,36]. Until now, several physiological or pathological factors were connected with the IL-6 level, including hormones, diet, exercise, and stress [37,38,39,40]. This study demonstrates for the first time that BCFA may also influence the expression level of *IL-6* in adipocytes. It has been shown that IL-6 can promote the synthesis of C-reactive protein (CRP) [41,42]. Therefore, the level of iso-BCFAs may indirectly influence the level of CRP. This may explain why in our previous work we document the inverse correlation between serum CRP and iso-BCFA levels [12].

Incubation of visceral adipocytes with both types of BCFA (iso-, and anteiso-BCFA) resulted in the decreased expression level of *ALOX-15* (Figure 3a,b). The expression of various lipoxygenases (LOX) isoforms, including *ALOX-15*, were reported in human visceral adipose tissue [43,44]. LOX also plays an important role in obesity and obesity-induced consequences, such as inflammation and insulin resistance [45,46,47,48,49]. Enzymes that are encoded by *ALOX-15* generate various bioactive lipid mediators, such as eicosanoids, hepoxilins, lipoxins, and other molecules from various PUFA substrates [48,50,51]. For example, 15-lipoxygenase converts arachidonic acid (AA) into 15-hydroxyeicosatetraenoic acid (15(S)-HETE), a known pro-inflammatory molecule [52,53,54]. Studies have shown that the level and activity of LOX-15 are increased in mice on a high-fat diet and obese patients [35]. This study has shown for the first time that a reduced level of BCFA in obese subjects can be one of the possible molecular mechanisms of this phenomenon.

This study has shown that 12-MTA, an anteiso-BCFA, did not affect the expression level of cyclooxygenase 2 gene (*COX-2*), while 14-MPA caused a statistically significant reduction in expression, only at the highest experimental concentration (Figure 3a,b). *COX-2* generates pro-inflammatory mediators—prostaglandins (mainly PGE2) from AA. Studies have shown that the expression level of *COX-2* is elevated in the subcutaneous adipose tissue of obese humans, and that *COX-2* is involved in the development of obesity-associated adipose tissue inflammation and insulin resistance [55,56,57]. This study demonstrates for the first time that BCFA can modulate *COX-2* expression.

Based on the results presented in this study, it can be concluded that iso-BCFA can be another regulatory factor involved in regulating inflammation, mainly by decreasing the expression level of pro-inflammatory genes such as *COX-2*, *IL-6* and *ALOX-15*. Lower iso-BCFA levels observed in obese patients can aggravate inflammation in adipose tissue and increase the risk of obesity-related metabolic diseases. This supposition is supported by the fact that an increase in iso-BCFA levels in obese subjects that lost body weight after bariatric surgery was associated with decreased inflammation (assessed based on serum CRP levels) [13]. It can be speculated that iso-BCFA may find potential applications as protective agents against obesity-induced consequences. Since the main source of BCFAs for humans is the dietary intake of common dairy products, modifying the diet of obese patients for BCFA content could be beneficial in terms of hyperlipidemia and inflammation reduction. However, to date, human trials assessing the health effects of the supplementation of iso-BCFA are still lacking and animal data are very limited. Moreover, there are currently no recommendations for the daily intake of BCFA. Very little is known about the daily intake of BCFAs in humans. Based on the daily intake of dairy and beef products in the United States, the daily intake of BCFA has been estimated to range from 220 mg/day to 500 mg/day [58,59]. To the authors’ knowledge there are no such studies from other countries. Moreover, diet is not the only source of BCFA in humans. Some studies have shown that BCFAs can be produced by gut microbiota [60] or can by synthesized de novo in mammals [11,61].Therefore, further investigations are required.

In the present study, the effects of BCFA were evaluated in visceral adipocytes because this is the main adipose tissue that is associated with obesity-related diseases [62,63]. Taking into consideration that lipid metabolism can be very depot-dependent, to better understand the comprehensive effect of BCFA on lipid metabolism, adipocytes from other adipose tissue should be analyzed in further research.

The limitation of this study is the fact that the expression of genes was studied only on the mRNA levels and not on the protein levels. The commercially available primary pre-adipocytes that were purchased for our experiments can be cultured for a limited number of passages, and the number of cells that were obtained after the differentiation of adipocytes allowed only for mRNA experiments. However, there is strong evidence that the expression of studied genes is regulated on the level of transcription [21,64,65].

## 4. Conclusions

In conclusion, this study demonstrates for the first time that iso-BCFAs can decrease the expression level of genes that are involved in lipid synthesis (except for *FASN*) and genes that encode pro-inflammatory proteins in a dose-dependent manner. Based on the results presented in this study, one can suppose that the decreased level of iso-BCFA that was previously observed in obese subjects may contribute to dyslipidemia and inflammation. Further studies should evaluate whether iso-BCFA supplementation in obese patients would be beneficial.

## Figures and Tables

**Table 1:** Structural differences among straight-chain fatty acid, iso- and anteiso-BCFA. BCFA—branched-chain fatty acid.

Type of Fatty Acid	15 Carbon-Atom Fatty Acid
Straight-chain fatty acid	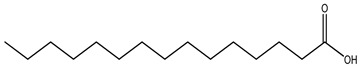 Pentadecanoic acid
Iso-BCFA	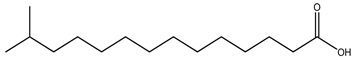 13-Methyltetradecanoic acid
Anteiso-BCFA	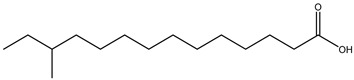 12-Methyltetradecanoic acid
